# Squamous Metaplasia Is Increased in the Bronchial Epithelium of Smokers with Chronic Obstructive Pulmonary Disease

**DOI:** 10.1371/journal.pone.0156009

**Published:** 2016-05-26

**Authors:** Helen M. Rigden, Ahmad Alias, Thomas Havelock, Rory O'Donnell, Ratko Djukanovic, Donna E. Davies, Susan J. Wilson

**Affiliations:** 1 Academic Unit of Clinical and Experimental Sciences, Faculty of Medicine, University of Southampton, Southampton, United Kingdom; 2 NIHR Southampton Respiratory Biomedical Research Unit, University Hospital Southampton, Tremona Road, Southampton, United Kingdom; H. Lee Moffitt Cancer Center & Research Institute, UNITED STATES

## Abstract

**Aims:**

To quantify the extent of squamous metaplasia in bronchial biopsies and relate it to the presence of chronic obstructive pulmonary disease (COPD), a smoking-related pathology.

**Methods:**

Bronchial biopsies (n = 15 in each group) from smokers with COPD GOLD stage1 and GOLD stage2, smokers without COPD and healthy non-smokers were stained immunohistochemically with a panel of antibodies that facilitated the identification of pseudostratified epithelium and distinction of squamous metaplasia and squamous epithelium from tangentially cut epithelium. The percentage length of each of these epithelial phenotypes was measured as a percent of total epithelial length using computerised image analysis. Sections were also stained for carcinoembryonic antigen and p53, early markers of carcinogenesis, and Ki67, and the percentage epithelial expression measured.

**Results:**

The extent of squamous metaplasia was significantly increased in both COPD1 and COPD2 compared to healthy smokers and healthy non-smokers. The amount of fully differentiated squamous epithelium was also increased in COPD1 and COPD2 compared to healthy non-smokers, as was the expression of carcinoembryonic antigen. These features correlated with one other.

**Conclusion:**

In subjects with COPD there is a loss of pseudostratified epithelium accompanied by an increase in squamous metaplasia with transition into a fully squamous epithelium and expression of early markers of carcinogenesis.

## Introduction

Squamous metaplasia (SQM) is a pre-neoplastic change of the bronchial epithelium observed in the lungs in response to toxic injury induced by cigarette smoke [1–4]. It is part of a multi-stage process [5–7] which may eventually lead to full neoplastic transformation, i.e. bronchial carcinoma. Not all SQM lesions progress to a neoplasia, particularly if low grade and some may regress to a normal epithelium [8–10], especially after smoking cessation [11].

Initially, during SQM quiescent basal cells within the pseudostratified epithelium re-enter the cell cycle and become hyperproliferative. During the next stage of the process, the epithelium begins to express markers of a squamous phenotype rather than those of the normal pseudostratified epithelium. These include squamous epithelial cytokeratins (CK) [5,6,12–14] and the cell adhesion molecule SQM1 [15]. Finally, when fully differentiated, having a squamous cell morphology, cells will express involucrin, a marker of terminal differentiation [16].

A history of cigarette smoking is associated with 90% of lung cancers with 15% of lifetime smokers developing lung cancer [17–20]. Chronic obstructive pulmonary disease (COPD) is also associated with smoking and is an independent risk factor for developing lung cancer, the risk being increased by up to 4.5 fold [21–26]. Between 50 and 70% of subjects with lung cancer also have COPD [18,27]. The cause of this increased susceptibility in subjects with COPD is unknown. Several possibilities have been suggested, including common molecular pathways [28,29], impaired ability to clear carcinogens due to obstructive airways [30] and ongoing chronic inflammation within the airways [27,31].

**Fig 1:**
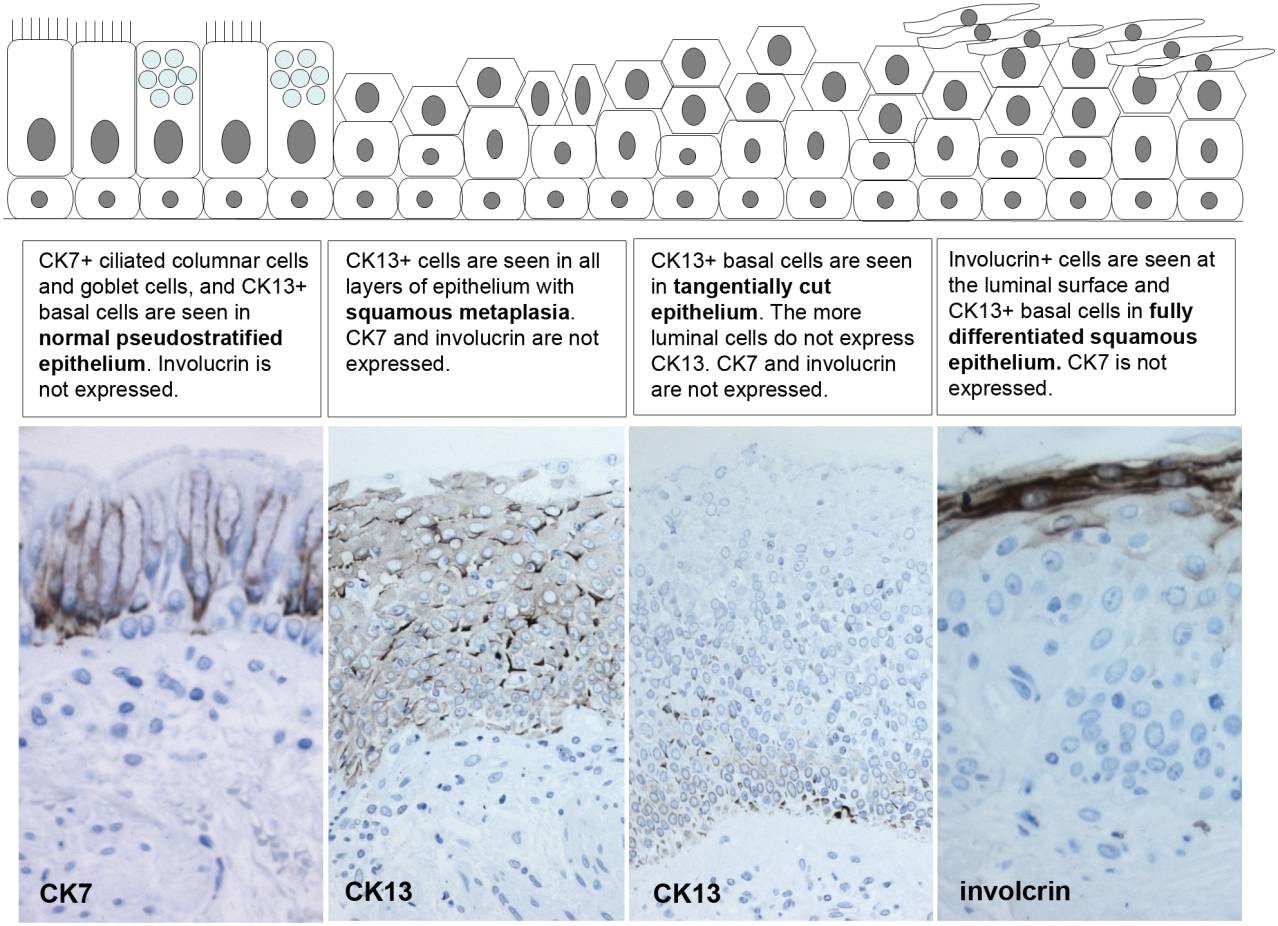
Cytokeratin and involucrin expression in the bronchial epithelium during transition from a normal pseudostratified epithelium to a squamous phenotype. Diagram, with matching photographs from bronchial biopsies included in this study, illustrating how the expression of CKs and involucrin (brown) changes as the normal pseudostratified bronchial epithelium undergoes transition towards a squamous phenotype.

SQM is observed in the bronchial epithelium of smokers [11], but to date there have been, to our knowledge, no studies to quantify it and relate it to the coexistence and severity of COPD. We have previously identified a panel of antibodies, CK7, CK13 and involucrin, that are suitable for identification and distinction of SQM and squamous epithelium in endobronchial biopsies, from tangentially cut epithelium [32], which is difficult based on morphology alone in small biopsies. CK7 is seen in luminal cells of the pseudostratified epithelium and its expression is lost during SQM and absent in squamous epithelium. CK13 expression is restricted to the basal cells of pseudostratified epithelium but is observed throughout the epithelium with SQM or a squamous phenotype. Involucrin is restricted to cells with a fully differentiated squamous morphology. This staining pattern is summarised in Fig 1 in the results.

The aim of the current study was to quantitate, using the above panel of antibodies, the amount of SQM and squamous epithelium (SE) within the bronchial epithelium of smokers with and without COPD, compared to healthy controls. Additionally, we have looked at the expression of the proliferation marker, Ki67, and the early markers of carcinogenesis, carcinoembryonic antigen (CEA) and p53. The relationship to severity of COPD and smoking history was also investigated.

## Materials and Methods

### Subjects and study design

This study used previously collected glycol methacrylate embedded bronchial biopsies from four subject groups (n = 15 in each group); healthy non-smokers, healthy smokers, and COPD subjects classified according to the GOLD guidelines [33] as having COPD stage 1 airflow obstruction (COPD1) and COPD stage 2 airflow obstruction(COPD2). The clinical characteristics of each subject were fully characterised (Table 1); data from some of these subjects has been reported previously [34].

**Table 1:** Subject characteristics.

Group	Gender	Age (years)	FVC / FEV_1_	FEV_1_ (% predicted)	Smoking history (pack years)
Healthy non-smokers	6M:9F	53.6 (8.2)	77.60 (4.88)	108.67 (12.20)	zero
Healthy smokers	5M:10F	48.2 (9.5)	78.80 (5.24)	100.53 (9.37)	36.73 (13.09)
COPD 1	12M:3F	56.7 (7.5)	65.13 (4.41)	91.53 (5.93)	44.00 (15.97)
COPD 2	10M:5F	56.7 (8.2)	60.87 (7.09)	66.60 (7.53)	50.33 (12.38)

Data are means and (standard deviations)

This study was approved by National Research Ethics Service Committee South Central—Southampton B (11/SC/0103) and the original biopsy collection for which subjects gave written informed consent by Southampton and South West Hampshire Research Ethics Committee (276/99 and 09/H0502/91).

### Immunohistochemistry

Two-micron sections were cut and stained immunohistochemically using an avidin-biotin technique and a panel of antibodies we have previously validated that facilitate the distinction and identification of epithelium with squamous metaplasia and fully squamous phenotype from tangentially cut epithelium in biopsy samples [32](Fig 1). This included mouse monoclonal antibodies to cytokeratin (CK) 7, CK13 and involucrin. We also used mouse monoclonal antibodies to pan-CKs (as a positive control), the proliferation marker Ki67 and to the early carcinogenesis markers p53 and carcinoembryonic antigen (CEA). Negative control sections were incubated with Tris-buffered saline (TBS) in replacement for the primary antibody. Further antibody details are shown in Table 2.

**Table 2:** Panel of antibodies used.

Antibody	clone	Catalogue number	Supplier	Working concentration
CEA	II-7	M7072	Dako, Ely, UK	0.2 ug/ml
CK7	OV-TL 12/30	M7018	Dako, Ely, UK	0.256 ug/ml
CK13	KS-1A3	C0791	Sigma, Poole, UK	4.94 ug/ml
Pan CK	C-11, PCK-26, CY-90, KS-1-A3, M20, A53-B/A2	C2562	Sigma, Poole, UK	dilution1:4000*
Involucrin	SY5	MS-126-P1	ThermoFisher Scientific, Runcorn, UK	0.5 ug/ul
Ki67	MIB-1	M7240	Dako, Ely, UK	0.102 ug/ml
P53	DO-7	NCL-L-p53-DO7	Novocastra, Leica Biosystems, Newcastle, UK	0.875 ug/ml

* concentration unavailable

The percentage lengths of epithelium that were either pseudostratified, were undergoing squamous metaplasia or were fully differentiated squamous epithelium, based on the patterns of cytokeratin and involucrin expression, were measured as a percent of total epithelial length (excluding tangentially cut) with the assistance of computerised image analysis (Zeiss KS400, Image Associates, Bicester, UK). The percentages of the epithelium staining for CEA as area ratio and the percentage nuclear epithelial area expression of p53 and Ki67, based on the red/green/blue colour composition of the DAB staining [35] within the intact epithelium, were assessed by computerised image analysis.

### Statistical analysis

Non-parametric statistical tests were used to analyse the results of the immunohistochemical analysis and data presented as medians and interquartile ranges. The Kruskall-Wallis ANOVA test was used to test for differences between the 4 groups and, where appropriate, the Mann Whitney U test was used for further analyses, with p<0.05 considered statistically significant. The Spearman Rank test was used to test for correlations between immunohistochemical data and clinical parameters.

## Results

Staining with CK7, CK13 and involurin enabled us to identify normal pseudostratified epithelium, that was undergoing squamous metaplasia and that had had undergone transition to a fully squamous epithelium (Fig 1). In normal pseudostratified epithelium the columnar and goblet cells were CK7+ and the basal cells CK13+. In squamous metaplasia CK13+ cells were seen throughout epithelium. In fully differentiated squamous epithelium involucrin+ cells were observed at the luminal surface (Fig 1).

**Fig 2:**
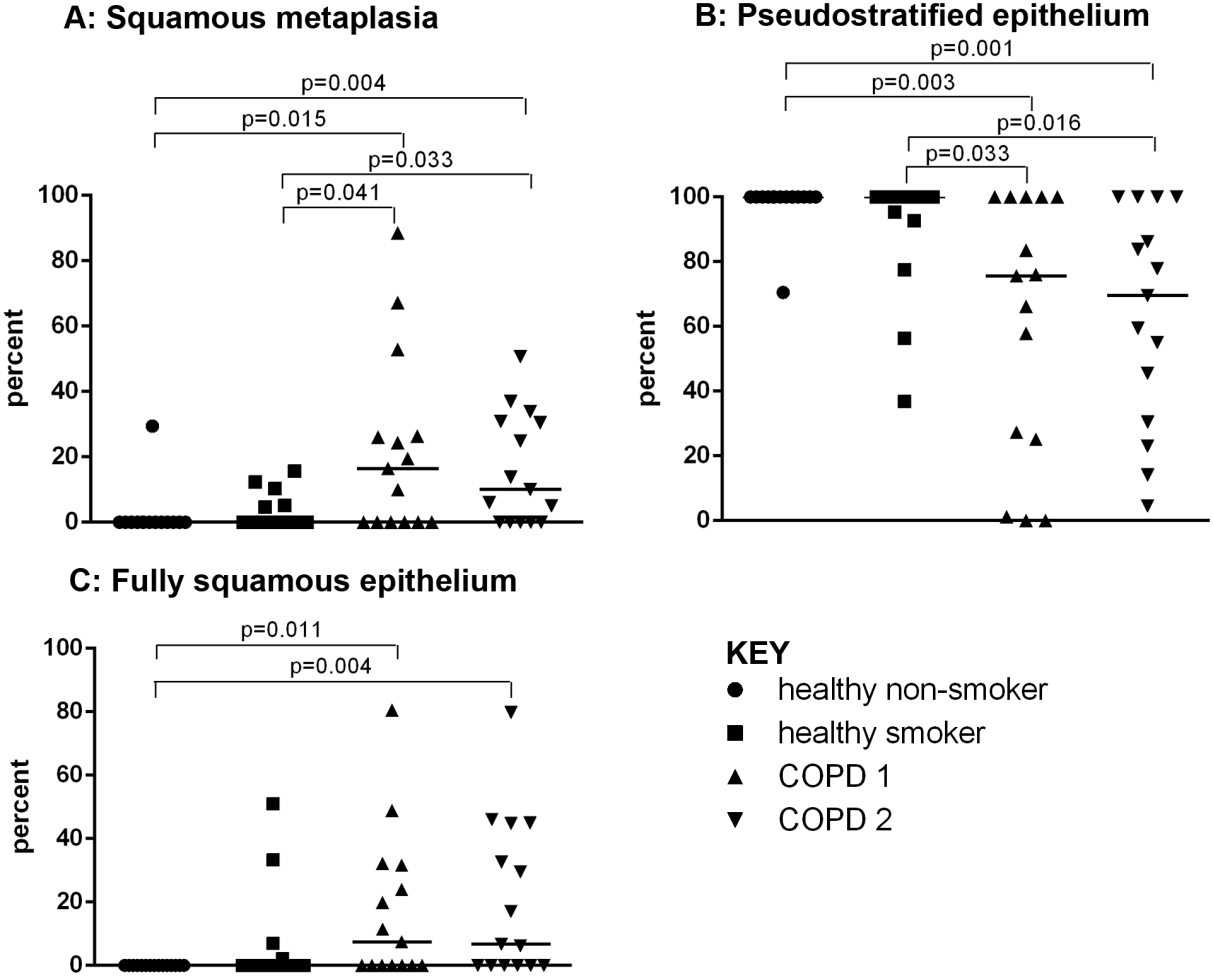
Graphs showing epithelial phenotype. The percentage of epithelium with squamous metaplasia (A), that with a pseudostratified (B) and a fully squamous (C) phenotype are shown in healthy non-smokers (●), healthy smokers (■) and subjects with COPD1(▲) and COPD2(▼). Significant differences between the groups are indicated.

A mean length of 2.04mm of epithelium was assessed for each subject. The extent of squamous metaplasia was increased in both COPD1 and COPD2, 16.5% (0–23.4%) & 10.0% (0–30.9%), respectively, when compared to healthy smokers (0% (0–5.2%)(p = 0.041 & p = 0.033) and healthy non-smokers (0%(0–0%))(p = 0.015 & p = 0.004)(Fig 2A). This was paralleled by a decrease in pseudostratified epithelium (Fig 2B). The amount of fully differentiated squamous epithelium was also increased in COPD1 (7.4% (0–32.7%)) and COPD2 (6.67%(0–44.8%)) compared to healthy non-smokers (0%(0–0%)), p = 0.011 and p = 0.004, respectively but not healthy smokers (Fig 2C).

There was a positive correlation between the extent of this squamous metaplastic and fully differentiated squamous epithelium and a corresponding negative relationship to the amount of pseudostratified epithelium as it is replaced by these altered phenotypes (Table 3).

**Table 3:** Epithelial correlations.

	Fully squamous epithelium	Pseudostratified epithelium	CEA expression	Ki67 expression
SQM Epithelium	Rho = 0.686 p<0.001	Rho = -0.909 p<0.001	Rho = 0.329 P = 0.010	Rho = 0.408 P = 0.001
Fully squamous epithelium	na	Rho = -0.885 p<0.001	Rho = 0.407 p = 0.001	Rho = 0.414 p = 0.001
Pseudostratified epithelium	na	na	Rho = -0.423 p = 0.001	Rho = -0.468 p<0.001

Spearman’s rho

**Fig 3:**
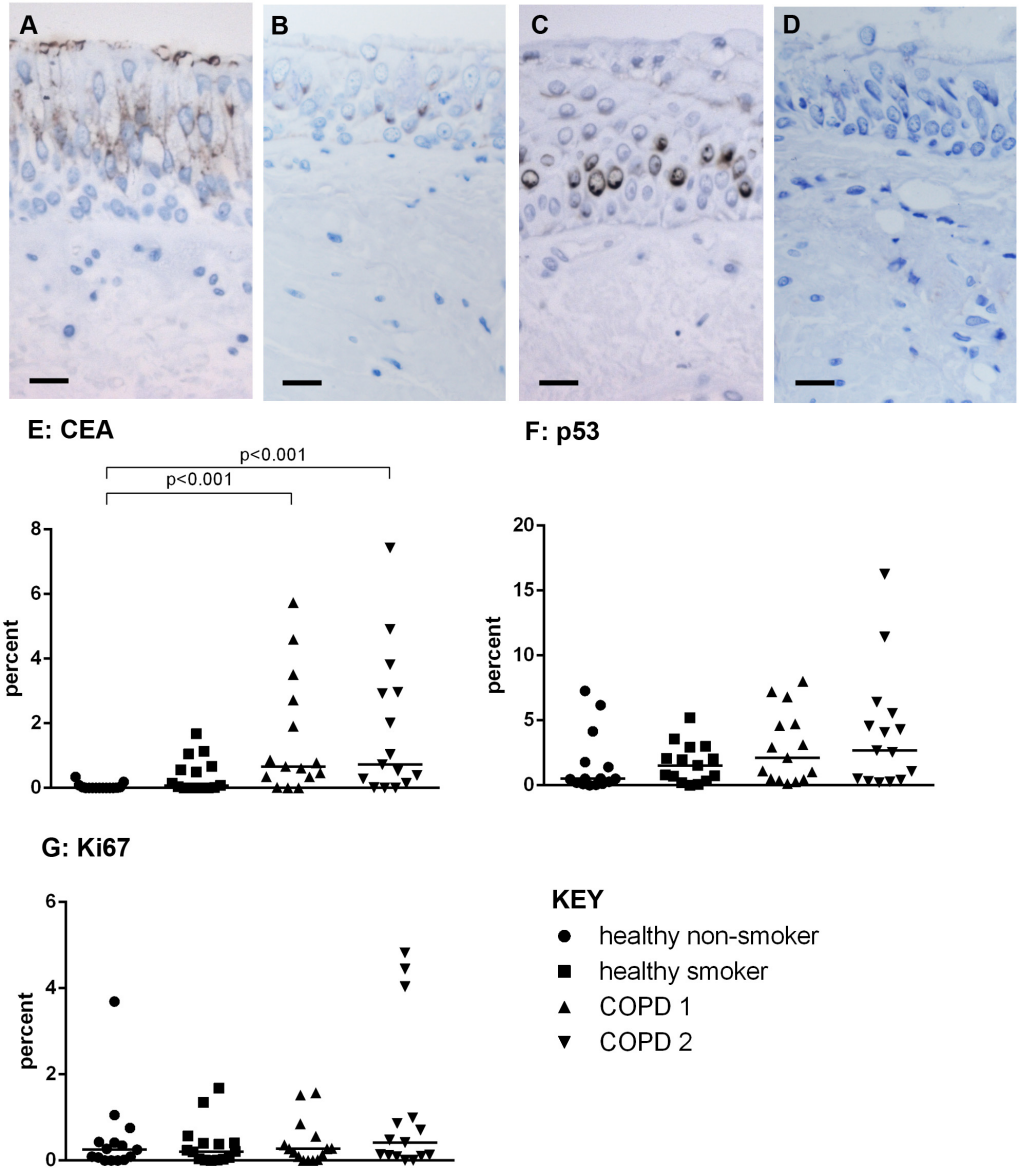
Epithelial expression of early markers of carcinogenesis and proliferation. CEA is expressed in the luminal cells of pseudostratified epithelium (A), p53 is weakly expressed in the perinuclear region of suprabasal cells (B), nuclear expression of ki67 can also be seen (C). Positive staining is brown. No staining was observed in the negative controls sections (D). Scale bar = 20 um. The percentage of the epithelium expressing CEA (E), p53 (F) and Ki67 (G) are shown in healthy non-smokers (●), healthy smokers (■) and subjects with COPD1(▲) and COPD2(▼). Significant differences and Kruskall Wallis P values between the groups are indicated.

Staining for CEA was observed in the luminal cells of the pseudostratified bronchial epithelium (Fig 3A) and was increased in COPD1 (0.7% (0.3–2.7%)) and COPD2 (0.7%(0.1–3.0%)) compared to healthy non-smokers (0%(0–0%))(p<0.001 for both groups)(Fig 3E). P53 was weakly expressed in the perinuclear region of cells (Fig 3B), Ki67 staining was also nuclear (Fig 3C). There was no difference in the expression of p53 or Ki67 across the groups (KW p = 0.14 and p = 0.52, respectively)(Fig 3F and 3G)).

The expression of both CEA and Ki67 had a positive relationship with the amount of epithelium with squamous metaplasia and that with a fully differentiated squamous phenotype and a negative relationship with pseudostratified epithelium. There were no correlations with p53 (Table 3).

The epithelial phenotype and expression of CEA and Ki67correlated with lung function but not with smoking history (Table 4). There was a negative relationship between squamous metaplasia, the amount of fully squamous epithelium, CEA expression with both Forced Expiratory Volume in 1 second (FEV_1_) and Forced Vital Capacity (FVC) /FEV_1_ and a positive relationship between the amount of pseudostratified epithelium and lung function. Ki67 had a negative correlation with FEV_1_ only.

**Table 4:** Correlations between epithelial phenotype and measures of lung function.

	SQM epithelium	Fully squamous epithelium	Pseudostratified epithelium	CEA expression	Ki67 expression
FVC/FEV_1_	rho = -0.49 p<0.001	rho = -0.44 p = 0.001	rho = 0.51 p<0.001	rho = -0.40 p = 0.001	ns
FEV_1_	rho = -0.42 p = 0.001	rho = -0.40 p = 0.002	rho = 0.44 p<0.001	rho = -0.45 p<0.001	Rho = -0.298 p = 0.021

Spearman’s rho

## Discussion

This study demonstrates increased expression of epithelial markers that are consistent with a squamous metaplastic phenotype, together with a loss of normal pseudostratified epithelium, in smokers with COPD when compared to both healthy smokers and healthy non-smokers. Furthermore an increase in the amount of epithelium with a fully differentiated squamous phenotype is observed in smoking COPD patients when compared to healthy non-smokers (Figs 1 & 2). These changes are accompanied by increased expression of the early carcinogenesis marker, CEA.

To our knowledge this is the first study assessing the extent of SQM within the bronchial epithelium of smokers with and without COPD and healthy control participants. Cosio *et al* [1] have reported increased SQM in the small airways of subjects with FVC/FEV_1_ ratios in the COPD range compared to those with lung function in the normal range. Together these data suggest that SQM can occur throughout the airways in response to cigarette exposure.

SQM has been observed to be increased in the bronchial epithelium of COPD patients who are current smokers when compared to those who are ex-smokers [11] and to be related to the intensity of smoking (number of cigarettes smoked in a day), but not pack year history [3]. In our study, all of the COPD subjects were current smokers and we also did not observe any relationship with pack-year history. We found no difference in SQM between healthy non-smokers and healthy current smokers, suggesting that COPD is a contributing factor to these SQM changes rather than smoking alone. The correlation of SQM with both FVC/FEV_1_ and FEV_1_ may suggest that similar mechanisms are driving the loss of lung function and the development of SQM. This is supported by the study of Araya *et al* [36] who linked SQM with evidence of fibroblast activation and correlated this with small airway wall thickening in COPD and disease severity.

In our study transition to a fully differentiated squamous epithelium, indicated by positive staining for involucrin in epithelium with a squamous phenotype, was higher in subjects with COPD1 and 2 compared to healthy non-smokers but not compared to healthy smokers. This suggests this is a consequence of both smoking and COPD. This concurs with the study by Araya [36] in which increased intensity of involucrin staining was reported in samples obtained during surgery for lung cancer in patients who also had stage 2 and 3 COPD, when compared to healthy organ donor lungs.

A possible mechanism for this shift towards a squamous epithelial phenotype is via activation of the epidermal growth factor (EGF) / EGF receptor (EGFR) pathway due to the oxidative stress induce by cigarette smoke [37]. Shakhiev *et* al have demonstrated that EGF in air liquid interface cultures skews the epithelium to differentiate towards a squamous phenotype with the expression of cytokeratin 14 and involucrin [37]. We have previously demonstrated upregulation of EGFR in conjunction with goblet cell hyperplasia in these subjects, but did not investigate the relevance to the presence of SQM [38].

We also studied the expression of CEA and p53 markers of early carcinogenesis and the expression of the cell proliferation marker Ki67.

The expression of CEA in the bronchial epithelium was increased in subjects with COPD 1 and 2 when compared to healthy non-smokers and correlated with the extent of SQM and a fully differentiated squamous epithelium. Previous studies have found increased CEA levels in serum and secretions of healthy smokers and subjects with COPD compared to healthy non-smokers [39], but no difference with COPD severity [40], suggesting raised CEA is a marker of smoking rather than the presence of COPD. However, our data and that of Athanassiadou [13], have demonstrated that CEA expression is related to the pre-neoplastic change of SQM, suggesting that it could be a useful marker of this pre-neoplastic change.

We did not observe any differences in the expression of either p53 or Ki67 across our subject groups. P53, a tumour suppressor gene, expression is not observed in normal cells [41] but is reported to be expressed in areas of epithelium with SQM [42–44] increasing as the epithelium becomes more dysplastic [42,43,45,46]. Ki67 is also reported to be increased in areas of SQM, and to be of predictive value in the progression to lung cancer [44]. To our knowledge none of our subjects had lung cancer or a history of lung cancer so the pre-neoplastic changes we have observed may be early events in this transition process, and these markers may have yet to be upregulated. Also, we only assessed expression in areas of epithelium with a normal pseudostratified appearance we did not assess expression in areas of SQM which may have been more appropriate. However, we did observe a significant correlation between Ki67 expression and the extent of epithelium that had a SQM and a fully squamous phenotype. Therefore future work assessing the expression in SQM and epithelium with a fully squamous phenotype is warranted.

SQM is a reversible pre-neoplastic change in the epithelium [8–10]. On long-term cessation of smoking, Lapperre *et al* [11] report a reduction in SQM in patients with COPD when compared to current smokers with COPD. Cessation also reduces the risk of progression to lung cancer [47, 48], as does corticosteroid therapy [49]. Hence early detection of these pre-neoplastic changes is important to facilitate early intervention. This is a challenge as at this early stage patients are asymptomatic, only presenting when progression to lung cancer has occurred. Autofluorescence bronchoscopy facilitates earlier detection of pre-invasive lesions [10,50–52] especially if used with appropriate biomarkers and histological assessment. This may be a strategy for monitoring high risk patients.

In conclusion we demonstrated that there is a loss of the normal pseudostratified epithelium in smokers with COPD together with pre-neoplastic changes evidenced by increased presence of SQM and fully differentiated squamous epithelium, together with expression of CEA. This may warrant aggressive smoking cessation strategies, with long-term follow up to assess regression to a normal phenotype or progression to lung cancer.
